# Non-aureus Staphylococci Species in the Teat Canal and Milk in Four Commercial Swiss Dairy Herds

**DOI:** 10.3389/fvets.2019.00186

**Published:** 2019-06-12

**Authors:** Julia Traversari, Bart H. P. van den Borne, Claudio Dolder, Andreas Thomann, Vincent Perreten, Michèle Bodmer

**Affiliations:** ^1^Vetsuisse Faculty, Clinic for Ruminants, University of Bern, Bern, Switzerland; ^2^Business Economics Group, Wageningen University, Wageningen, Netherlands; ^3^Vetsuisse Faculty, Institute of Veterinary Bacteriology, University of Bern, Bern, Switzerland

**Keywords:** intramammary infection, teat canal, non-aureus staphylococci, species distribution, mastitis

## Abstract

Non-aureus staphylococci (NAS) are frequently found in milk samples as well as on the teat apex and in the teat canal and are known to be a cause of subclinical mastitis. The objective of this study was to investigate the relationship between NAS species colonizing the teat canal and those causing intramammary infection (IMI) in four commercial dairy herds. Teat canal swabs were obtained and thereafter milk samples were aseptically collected and evaluated for the presence of staphylococci using selective agar plates. Species identification was performed using matrix-assisted laser desorption/ionization time–of–flight mass spectrometry. The relationship between NAS species distribution and sample type (teat canal vs. milk samples) was quantified using hierarchical multivariable logistic regression models. The most prevalent NAS species in teat canal swabs were *S. xylosus* (35%), *S. vitulinus* (10%), and *S. chromogenes* (7%), whereas in milk samples *S. chromogenes* (5%), *S. xylosus* (5%), and *S. haemolyticus* (4%) were most prevalent. There were significantly higher odds for *S. vitulinus* (OR = 215), *S. xylosus* (OR = 20), *S. sciuri* (OR = 22), *S. equorum* (OR = 13), and *S. succinus* (OR = 10) to be present in teat canal swabs than in milk samples. Differences between herds in NAS species distribution were found and were most pronounced for *S. succinus* and a *S. warneri*-like species. This information aids in the understanding of NAS species as an etiology of IMI and should be taken into account when interpreting milk culture results.

## Introduction

Non-aureus staphylococci (NAS) are frequently isolated from bovine milk samples (1–3). They are one of the main causes of subclinical mastitis in dairy herds that have successfully controlled major mastitis pathogens (1, 4) such as *Staphylococcus aureus, Streptococcus uberis, Streptococcus agalactiae, Streptococcus dysgalactiae*, and coliforms (5).

Non-aureus staphylococci species considered most relevant as causes of intramammary infection (IMI) are *S. chromogenes, S. epidermidis, S. haemolyticus, S. simulans*, and *S. xylosus* (6). Ecological, epidemiological and virulence behavior, as well as the antimicrobial resistance profile, vary widely between species (6–10) and even amongst strains of a given species (3, 11–14).

Multiple studies demonstrate that NAS are the most prevalent bacteria found on teat apices of lactating dairy cows (8, 15). They are also frequently isolated from teat canal samples (16–18). These findings suggest that teat apex and canal colonization may act as a reservoir for NAS species causing IMI. The importance of the teat canal as a barrier against bacterial invasion was partly supported by two recent studies which showed that, collectively, NAS were more frequently found in samples obtained by milking the quarter than in samples collected directly from the udder cistern or using the cannula technique (19, 20).

To the authors' knowledge, only one study has investigated the relationship between teat canal colonization and IMI on a NAS species level (17). Further studies to assess the extent of teat canal colonization and IMI by NAS species are needed to gain more insight into their ecology and epidemiology. Therefore, the objective of this study was to investigate the relationship of NAS species identified in milk with those colonizing the teat canal.

## Materials and Methods

The study was approved by the committee for animal experimentation (KTV) of the Canton of Bern (BE58/14), Switzerland.

### Selection and Characterization of Study Herds

Four commercial Swiss dairy herds (A, B, C, and D) were enrolled in the study. Herd health visits were part of the clinical service of the Clinic for Ruminants, University of Bern, and took place every 2 weeks. During these regular herd health visits, California Mastitis Tests (CMT) (21) were performed on cows with an individual composite SCC > 150,000 cells/ml, as indicated by the Swiss law (22). Quarters scoring > 1 in the CMT for the first time were sampled for bacteriological culture, species identification was subsequently performed using matrix-assisted laser desorption/ionization time–of–flight mass spectrometry (MALDI–TOF MS). Herds A, B, and C were also part of a longitudinal 1-year study to determine risk factors for NAS species IMI (23). Data collected during the herd health visits and in the aforementioned study were utilized to select herds with a known NAS status for this study. The proportion of samples NAS-positive compared to all quarter samples taken in 2013 were: A = 35.7%, B = 42.9%, C = 33.3%, D = 39.1%). Moreover, the herds had a low proportion of IMI caused by major mastitis pathogens (<20% of samples).

On farm A and B, cows were housed in free-stall barns with straw-bedded cubicles, while cows on farm C and D were housed in a tie-stall bedded on rubber mats covered with straw and sawdust, respectively. Milking was performed twice daily by one milker. For teat cleaning, all farmers used at least one disposable disinfection towel per cow. Post-milking teat disinfection with iodine-based products was performed routinely after each milking on farm A and D, sporadically (i.e., for high cell count cows) on farm B, and never on farm C.

### Sampling Procedure

Herds were visited once in spring/summer 2015. Individual quarter milk samples and teat canal swabs were collected aseptically from all lactating cows. Teat cleaning and, if necessary, udder cleaning was performed by the milkers prior to sampling. Teats were then thoroughly disinfected with cotton swabs drenched in ethanol (70%). A sterile swab (Transwab ENT Amies Plain, MWE Medical Wire & Equipment, Corsham, Wiltshire, England) was inserted approximately 3 mm into the teat canal, rotated tree times and extracted. Subsequently, pre-milking of at least three streams of milk was performed, teat ends were disinfected again and individual quarter foremilk samples were aseptically collected. Latex gloves were worn during the whole procedure and changed after each cow. The milk samples were transported to the laboratory within 40 min at a maximum temperature of 7°C and were then frozen at −20°C until further analysis (1–3 days after sampling). Clinical mastitis cases were excluded from the study.

### Laboratory Analysis

Milk samples were thawed and centrifuged at 590 × g for 10 min at 20°C. Ten microliter of milk sediment and the teat canal swabs were directly streaked on chromogenic agar plates selective for staphylococci (SaSelect, Bio-Rad, Marnes**-**la**-**Coquette, France) and incubated at 37°C for 48 h. Growth of staphylococci from both milk and teat canal swabs on the selective agar was previously validated as part of a parallel study (23). Staphylococci were differentiated from non–staphylococci species, such as *Corynebacterium spp., Bacillus spp., and Aerococcus spp*., based on colony shape and color. All suspected staphylococci isolates were identified to the species level by selecting one colony from each phenotypically different group on the plate and using MALDI–TOF MS (Microflex LT, Bruker Daltonics GmbH, Bremen, Germany). The extraction technique using 70% formic acid and α-Cyano-4-hydroxycinnamic acid (HCCA)-Matrix (Bruker Daltonics GmbH, Bremen, Germany) was applied. Isolates with scores ≥2.0 were identified to the species level according to manufacturer's guidelines. In a previous study, we identified two types of isolates displaying a score below 2.0 by MALDI–TOF MS (23). These isolates were found to be related to *S. warneri* and to *S. devriesei* by 16S rRNA gene-sequencing as well as by sequencing of the *rpoB* or *dnaJ* genes and were considered as *S. warneri*-like and *S. devriesei*-like, respectively (23). The reference spectra of these two species were introduced into the MALDI-TOF MS database and the updated database was used for the current study.

Teat canal swabs and milk samples with more than three different NAS species on the selective agar plates were considered as mixed staphylococcal species (23, 24). Incidentally growing non-staphylococci species were not considered for further analysis.

### Definition of IMI

To maximize sensitivity, quarters were defined as having a NAS IMI if ≥100 CFU (colony forming units) per milliliter of at least one NAS species from a single sample were identified, following Dohoo et al. (25) and De Visscher et al. (26). Accordingly, teat canals were considered to be colonized if ≥1 CFU of at least one NAS species was identified.

### Statistical Analyses

Raw data were entered and stored in Excel (Microsoft Excel 2010). Data management and statistical analyses were performed using the melogit command in STATA 14 (StataCorp, College Station, Texas, USA).

The bacteriological status “mixed staphylococci species” was analyzed first. The hierarchical logistic regression model for the probability of the bacteriological status “mixed staphylococci species” was as follows:

$$\begin{array}{l} \text{logit }(“\text{mixed staphylococci species}”)\;=\text{ intercept }+\text{ sample}\\ \text{type }+\text{ herd }+\text{ sample type x herd }+\text{ u }+\text{ v }+\text{ error} \end{array}$$

where sample type included teat canal swab vs. milk sample, herd represented the herd (A, B, C, and D) in which mixed staphylococci species were identified to allow for between-herd differences in NAS-species distribution (26–29), and u and v represented the random intercepts for quarter and cow. Covariates in the model were evaluated by the Type 3 test.

The “mixed staphylococci species” observations were subsequently excluded from the model in order to evaluate the NAS species individually. The same statistical model was applied for analyzing the NAS species but the sample type x herd interaction term was excluded because it was non-significant for all NAS species evaluated. The random quarter effect had to be removed from the model for *S. chromogenes* because of non-convergence. Staphylococci species with ≥5 observations in milk samples and swabs in total were analyzed individually, the remaining species were grouped and analyzed as “Others.”

The proportion of explained variance for species-specific NAS IMI at quarter and cow level was estimated based on the final NAS species-specific multilevel logistic regression model. Using the latent variable approach, by assuming that the variance at sample level was π^2^/3 (30), the total variance ($\sigma_{total}^{2}$) was estimated to be:

$$\begin{array}{r} \sigma_{total}^{2}=\sigma_{cow}^{2}+\sigma_{quarter}^{2}+\frac{\pi^{2}}{3} \end{array}$$

where $\sigma_{cow}^{2}$ is the variation occurring at cow level and $\sigma_{quarter}^{2}$ the variation at quarter level.

## Results

Milk and teat canal samples of 97 lactating Red Holstein (including Red Factor Black Holstein) and Swiss Fleckvieh cows (herd A: *n* = 27, herd B: *n* = 15, herd C: *n* = 19, herd D: *n* = 36) were included in the analysis.

### Mixed Staphylococci Species

**Figure 1:**
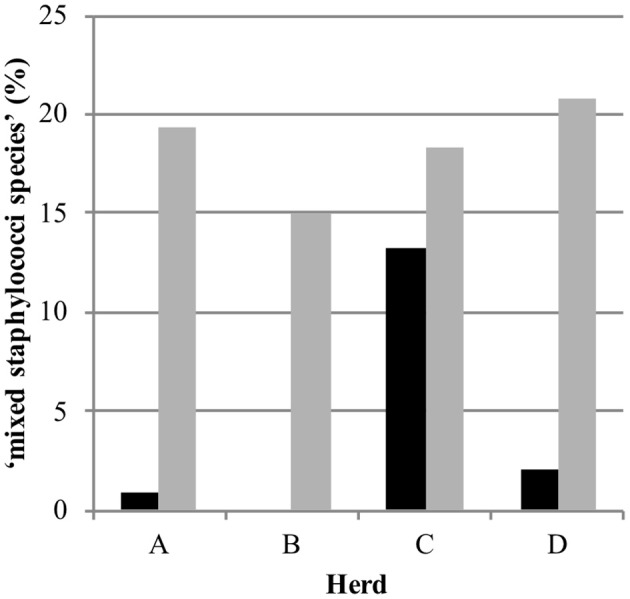
Distribution of the observed bacteriological status “mixed staphylococci species” in 387 quarter milk samples (black) and 383 quarter teat canal swabs (gray) in four commercial dairy herds (herds A, B, C, and D) in Switzerland.

Out of 770 quarter milk and teat canal samples, 11.3% (*n* = 87) contained mixed staphylococci species, consequently 88.7% (*n* = 683) of all samples contained zero to three staphylococci species. The final statistical model evaluating the bacterial status “mixed staphylococci species” identified a significant interaction between sample type and herd (Table 1). Mixed staphylococci species were significantly more frequently identified in teat canal swabs compared to milk samples. This was observed in all herds, except in herd C where a similar distribution was found (Figure 1).

**Table 1:** Final hierarchical logistic regression model evaluating the presence of mixed non-aureus staphylococci species in 387 quarter milk samples and 383 quarter teat canal swabs in four commercial dairy herds in Switzerland.

**Variable**	**Category**	**Frequency (*N*)**	**Prevalence (%)**	**Regression coefficient (95%-CI)**	***P*-value**
Intercept				−4.9 (−7.0–−2.9)	<0.001
Sample type	Swab	383	73 (19.1%)	3.4 (1.3–5.4)	0.001
	Milk	387	14 (3.6%)	Ref.^a^	
Herd	A	216	22 (10.2%)	Ref.	0.004
	B	120	9 (7.5%)	−0.3 (−1.3–0.7)	
	C	146	23 (15.8%)	2.9 (0.8–5.0)	
	D	288	33 (11.5%)	0.8 (−1.5–3.2)	
Sample type x herd	Swab in herd B			0.00	0.003
	Swab in herd C			−3.0 (−5.2–−0.7)	
	Swab in herd D			−0.7 (−3.1–1.6)	

a *Ref. = reference*.

*Variance of random quarter and cow-effect was 0 and 0.54, respectively*.

### NAS

Eighteen different NAS species were identified. The NAS species identified most often in both milk sample and teat canal of the same quarter was *S. chromogenes* (*n* = 12) (Table 2). As presented in Table 3, the most prevalent NAS species found in the teat canal was *S. xylosus* (34.8%, *n* = 108), followed by *S. vitulinus* (10.0%, *n* = 31) and *S. chromogenes* (7.1%, *n* = 22). The most prevalent NAS species found in milk was *S. chromogenes* (5.4%, *n* = 20), followed by *S. xylosus* (4.6%, *n* = 17), and *S. haemolyticus* (3.8%, *n* = 14).

**Table 2:** Distribution of non-aureus staphylococci in milk samples and teat canal swabs taken from the same quarter (*N* = 299) in four commercial dairy herds in Switzerland.

**Species**	**Frequency swab (%)**	**Frequency milk (%)**	**Frequency both milk and swab (%)**
		**N^a^**	**N**
*S. xylosus*	106 (35.5%)	15 (5.0%)	10 (3.3%)
*S. vitulinus*	25 (8.4)	2 (0.7%)	1 (0.3%)
*S. chromogenes*	19 (6.4%)	16 (5.4%)	12 (4.0%)
*S. haemolyticus*	17 (5.7%)	9 (3.0%)	1 (0.3%)
*S. sciuri*	15 (5.0%)	1 (0.3%)	-
*S. devriesei*-like	14 (4.7%)	8 (2.7%)	3 (1.0%)
*S. succinus*	12 (4.0%)	2 (0.7%)	-
*S. equorum*	10 (3.3%)	-	-
*S. warneri*-like	8 (2.7%)	11 (3.8%)	4 (1.3%)
*S. arlettae*	3 (1.0%)	1 (0.3%)	-
*S. simulans*	2 (0.7%)	2 (0.7%)	1 (0.3%)
*S. auricularis*	2 (0.7%)	-	-
*S. fleuretti*	2 (0.7%)	-	-
*S. epidermidis*	1 (0.3%)	2 (0.7%)	1 (0.3%)
*S. capitis*	1 (0.3%)	1 (0.3%)	-
*S. hominis*	1 (0.3%)	1 (0.3%)	-
*S. saprophyticus*	1 (0.3%)	1 (0.3%)	-
*S. agnetis*	-	1 (0.3%)	-

a *Number of positive samples*.

*Eighty-six quarters were excluded from the analysis because of missing teat canal swabs or milk samples*.

**Table 3:** Final multilevel multivariable logistic regression models describing the non-aureus staphylococci species distribution in teat canal swabs and milk samples (*n* = 683) in four commercial dairy herds in Switzerland.

					**Herd (vs. herd A)**			
	**Frequency (%)**		**Swab (vs. milk)**		**OR (95% CI)^b^**			
**Species**	**Swab (N^a^ = 310)**	**Milk (*N* = 373)**	**OR (95%-CI)**	***P*-value**	**B**	**C**	**D**	***P*-value**
*S. vitulinus*	31 (10.0%)	2 (0.5%)	215.6 (17.3–2690.1)	<0.001	22.1 (0.1–7019.8)	1367.3 (5.3–353767.1)	NI^c^	0.008
*S. sciuri*	16 (5.2%)	1 (0.3%)	22.5 (2.9–175.9)	0.003	NI	0.5 (0.1–2.9)	1.2 (0.3–3.9)	0.596
*S. xylosus*	108 (34.8%)	17 (4.6%)	20.3 (8.5–48.5)	<0.001	3.7 (1.3–10.5)	1.7 (0.6–4.7)	3.4 (1.4–8.1)	0.026
*S. equorum*	10 (3.2%)	1 (0.3%)	12.8 (1.6–101.2)	0.016	2.7 (0.7–10.1)	0.4 (0.0–3.4)	NI	0.104
*S. succinus*	12 (3.9%)	2 (0.5%)	9.6 (1.9–48.2)	0.006	Herd B only	-	-	-
*S. devriesei*-like	14 (4.5%)	12 (3.2%)	2.0 (0.7–5.4)	0.175	0.4 (0.0–3.5)	0.2 (0.0–1.9)	0.4 (0.1–2.2)	0.469
*S. chromogenes*	22 (7.1%)	20 (5.4%)	1.6 (0.8–3.3)	0.218	0.9 (0.1–12.9)	1.7 (0.2–16.9)	14.8 (2.5–88.7)	0.004
*S. haemolyticus*	17 (5.5%)	14 (3.8%)	1.5 (0.7–3.2)	0.259	1.0 (0.4–2.9)	0.2 (0.1–1.2)	0.6 (0.2–1.5)	0.253
Others^d^	13 (4.2%)	11 (2.9%)	1.5 (0.6–3.8)	0.356	4.6 (1.1–19.2)	0.9 (0.2–5.1)	0.8 (0.2–3.5)	0.067
*S. warneri*-like	8 (2.6%)	13 (3.5%)	0.6 (0.2–2.0)	0.402	-	Herd C only	-	-

a *N = Number of positive samples*.

b *OR (95% CI) = Odds ratio (95% Confidence interval)*.

c *NI = species not identified in this herd*.

d *Other NAS species: S. agnetis, S. arlettae, S. auricularis, S. capitis, S. epidermidis, S. fleuretti, S. hominis, S. saprophyticus, and S. simulans*.

*Up to three species per sample was allowed*.

The final statistical models evaluating NAS species distributions in milk samples and teat canal swabs are presented in Table 3. There were significantly higher odds for *S. vitulinus* (OR = 215.6), *S. xylosus* (OR = 20.3), *S. sciuri* (OR = 22.5), *S. equorum* (OR = 12.8), and *S. succinus* (OR = 9.6) to be present in teat canal swabs than in milk samples. There were no differences in occurrence of the species in question between milk samples and teat canal swabs for the other NAS species evaluated. Differences between herds concerning species distribution were observed for *S. chromogenes, S. succinus, S. vitulinus, S. warneri*-like, and *S*. *xylosus*.

### Variance Components

Variance components of all final models are presented in Table 4 and differed between the evaluated species.

**Table 4:** Variance component estimation at cow, quarter and sample level (teat canal swabs vs. milk) for all 11 final multilevel logistic regression models describing the non-aureus staphylococci species distribution in four commercial dairy herds in Switzerland.

	**Cow**		**Quarter**		**Sample**		**Total**	
	**Var. est.^a^**	**%**	**Var. est**.	**%**	**Var. est**.	**%**	**Var. est**.	**%**
*S. xylosus*	0.73	14.6	0.96	19.3	3.29	66.1	4.98	100
*S. vitulinus*	11.40	77.6	0	0	3.29	22.4	14.69	100
*S. equorum*	0	0	0	0	3.29	100.0	3.29	100
*S. sciuri*	0.77	19.0	0	0	3.29	81.0	4.06	100
*S. succinus*	6.14	65.1	0	0	3.29	34.9	9.43	100
*S. haemolyticus*	0.11	2.9	0.46	11.9	3.29	85.2	3.86	100
*S. devriesei*-like	3.71	40.5	2.16	23.6	3.29	35.9	9.16	100
*S. warneri*-like	15.40	71.3	2.91	13.5	3.29	15.2	21.59	100
*S. chromogenes*	3.29	50.0	NA^c^	NA	3.29	50.0	6.58	100
Others^b^	0.19	3.3	2.30	39.8	3.29	56.9	5.78	100

a *Variance estimation*.

b *Other NAS species: S. agnetis, S. arlettae, S. auricularis, S. capitis, S. epidermidis, S. fleuretti, S. haemolyticus-like, S. hominis, S. saprophyticus, S. sciuri and S. simulans*.

c *Not applicable*.

## Discussion

Although the relationship between NAS species causing IMI and those colonizing the teat canal has rarely been quantified (17), a difference in distribution of NAS species between teat canal samples and milk samples was expected. Previous studies found uneven distributions of different NAS species in milk samples and teat apices, including NAS species relevant and less relevant for IMI respectively (8, 16, 27, 31). The teat canal acts as a barrier against invading microorganisms causing IMI and several studies have shown that it harbors several different bacterial species, including *Staphylococcus spp., Enterobacteriaceae, Streptococcus spp*., and *Clostridium spp*. (17, 18, 32).

For some NAS species, no significant differences between occurrence in milk samples and teat canal swabs were found in this study, which is mirrored by the distribution of the NAS species found in both sample types of the same quarter. A possible interpretation is that the NAS species without a difference between sample types are more likely to cause IMI. This was previously reported for *S. chromogenes* and *S. haemolyticus* (11, 14, 29). Molecular analysis for strain characterization is needed to determine if the isolates found in teat canals and milk samples are identical. Temporal changes in NAS species occurrence were not assessed and it therefore remains unclear if they first colonized the teat canal and caused an IMI by invading the mammary gland or vice versa. It must be noted that the lack of statistical significance for some NAS species might be due to a low statistical power. However, given the low magnitude of these point estimates, as represented by the odds ratio, the difference between teat canal swabs and milk samples will not be clinically relevant for these NAS species.

For *S. chromogenes*, Quirk et al. (17) showed that teat canal colonization mostly preceded IMI, whereas the opposite was true for *S. xylosus*. Various studies agree on the host-associated characteristics of *S. chromogenes* since it is mainly isolated from cows' body-sites and milk and rarely from the environment (8, 16, 28). Strain typing showed high clonality and the capacity of *S. chromogenes* to disseminate within herds (13). Additionally, several authors confirmed its ability to cause persistent IMI (3, 29, 33). Some *S. chromogenes* strains are able to adhere to and enter bovine mammary epithelial cells (12) and a difference between pathogenicity and *in vivo* growth capacity was observed (14), which supports the hypothesis of host adaptation.

In the current study, *S. haemolyticus* was found in both milk samples and teat canal swabs, but rarely in both samples from the same quarter. The presence in both teat canal and milk samples confirms findings of other studies (8, 15, 17, 34). Presence on teat apices and in teat canals, however, seems to vary since *S. haemolyticus* was not found in teat canals in two separate studies by Gill et al. (18) and Taponen et al. (16). This difference might be due to the finding of Piessens et al. (28), who reported that the environment (air, slatted floor, sawdust) was the main reservoir for this species. A study from Leroy et al. (11) comparing subtypes from milk and teat apices indicated that IMI originated from strains present on the teat skin. Thus, *S. haemolyticus* appears to be a versatile species that can adapt to the animal (skin, teat canal, udder) as well as to the environment.

*Staphylococcus xylosus* was isolated more frequently in the teat canal than in milk samples and rarely in both samples from the same quarter, indicating that this species often colonizes the teat canal without necessarily causing an IMI. Together with *S. chromogenes, S. xylosus* is one of the most frequently identified NAS species in milk (6). The higher proportion of *S. xylosus* in teat canals compared to milk might be explained by a primarily environmental reservoir, as postulated by Piessens et al. (28); however, other authors have cultured the species from teat apex, udder skin, perineal tissue, and milkers' hands (16, 27, 31).

*Staphylococcus warneri*-like may represent a new species related to *S. warneri* (23) and was found in both samples from the same quarter in four of 19 cases, exclusively in study herd C. It also had a high within-herd occurrence (23) which may be a result of either a high incidence, a low cure rate, or both (3). Although not significant, this was the only NAS species found more frequently in milk samples than in teat canal swabs, which could be explained by a low cure rate and would be in accordance with Mørk et al. (3), who found that *S. warneri* can cause persistent IMI. Its predominant reservoir is unclear, but *S. warneri* has rarely been isolated from environmental samples (28) and is part of the human skin microbiota (35, 36).

In the present study, the odds to find *S. vitulinus* in the teat canal, rather than in milk, were the highest of all NAS species. To the authors' knowledge, *S. vitulinus* has not been previously found in teat canal samples but seems to be well-adapted to this particular microenvironment in the herds that we sampled. *Staphylococcus vitulinus* was rarely found to cause IMI (23, 26, 37) and was only recovered from a small proportion of teat apex samples (8, 27, 31). This might indicate that this species does not favor the mammary gland, but acts opportunistically.

Among the less prevalent species identified, *S. sciuri, S. equorum*, and *S. succinus* also had higher odds to be found in the teat canal vs. in milk samples. This was not the case for *S. devriesei*-like and “Others.” *Staphylococcus equorum* and *S. sciuri* were primarily isolated from the environment (28) and extramammary parlor-related habitats, such as clusters, liners, and milkers' gloves (8), but are also known to cause IMI (23, 28, 29). *Staphylococcus equorum* was among the most frequently found NAS species on teat apices (15, 27, 38) but has only been isolated once from the teat canal (17). Moreover, it was the predominant species in bulk milk in a Belgian longitudinal study (37). Quarters with dirty teat apices before calving were more likely to be infected with *S. equorum* and *S. sciuri* after parturition, supporting their environmental nature (26). *Staphylococcus succinus* was isolated from teat apices and rarely from the teat canal (16). *Staphylococcus devriesei* was frequently isolated from teat apices before parturition (38), but, to the authors' knowledge, not from the teat canal. *Staphylococcus devriesei, S. devriesei*-like, and *S. succinus* are able to cause IMI (23), but little is known about their reservoirs and epidemiology.

Variance estimates were the highest at cow level for *S. chromogenes, S. devriesei*-like, *S. succinus, S. vitulinus*, and *S. warneri*-like, indicating that a high proportion of the unexplained variance of IMI is determined by cow characteristics such as the efficiency of the innate non-local immune system. For *S. equorum, S. haemolyticus, S. sciuri*, and *S. xylosus* the biggest variance occurred at the sample level, which might be caused by sample location differences such as local barriers (i.e., the teat canal keratin layer) and the local immune system preventing a teat canal colonization to develop into IMI.

Some authors claim that certain NAS species might belong to the normal intramammary microbiota (39, 40) and may also play a role in the modulation of mastitis susceptibility, as recently reviewed by Derakhshani et al. (41). This concept, however, is questioned by others (42).

Mixed staphylococci species were found significantly more often in teat canal swabs samples (19.1%) than in milk samples (3.6%), except in herd C. The high presence of flies in this herd, as observed during the regular herd visits and the longitudinal study preceding the current study (23), might influence the bacteria population on teats and in teat canals since they may transmit bacteria causing IMI (43, 44). The same definition for mixed staphylococcal species was used in our above-mentioned previous study and also by Mahmmod et al. (24). In order to avoid preclusion of potentially relevant information, considering that the teat canal—similar to teat apices—harbors many different NAS species (8, 15–18) and taking into account the duality of its role—as a barrier against IMI on one side and potential reservoir for NAS species causing IMI on the other—the decision to include culture results with up to three *Staphylococcus* species was made.

The objective of this cross-sectional study was to assess the relationship between NAS species colonizing the teat canal and those causing IMI, thereby emphasizing the importance of *S. chromogenes, S. haemolyticus*, and *S. xylosus* as potential causative agents of IMI. In contrast, species such as *S. equorum, S. vitulinus*, and *S. succinus* seem to be more important as residents of the teat canal.

The small number of animals and herds sampled might limit the extrapolation of the results to the general Swiss dairy cattle population. Nevertheless,—despite not being longitudinal—this study provides new insights in the complex epidemiology and ecology of NAS. In light of the results and the previously mentioned dual role of the teat canal and the fact that NAS are frequently found on teat apices (8, 15), some relevant consequences arise. In particular, the difference in NAS distribution between sample types needs to be considered when interpreting milk culture results. This is highlighted by the fact that some NAS species were found significantly more often in teat canal swabs than in milk, which is line with other studies where isolation of NAS diminished if the teat canal was bypassed for milk sampling (19, 20). Consequently, the definition of IMI relying on culture results only is challenged, and consideration of milk SCC may be valuable in determining the causative role of NAS species (and other bacteria) isolated from conventionally collected milk samples.

## Data Availability

Datasets are available on request. The raw data supporting the conclusions of this manuscript will be made available by the authors, without undue reservation, to any qualified researcher.

## Ethics Statement

This study was carried out in accordance with the recommendations of the committee for animal experimentation (KTV) of the Canton of Bern (BE58/14), Switzerland.

## Author Contributions

BvdB and MB conceived and designed the study. JT and CD performed sample collection and part of the bacteriological analyses. AT and VP conceived and supervised the bacteriological part of the study. JT and BvdB analyzed the data and interpreted the study results together with MB. JT drafted the manuscript. BvdB and MB performed manuscript review. All authors contributed to manuscript revision and have read and approved the final manuscript.

### Conflict of Interest Statement

The authors declare that the research was conducted in the absence of any commercial or financial relationships that could be construed as a potential conflict of interest.
